# Direct reconstruction for simultaneous dual-tracer PET imaging based on multi-task learning

**DOI:** 10.1186/s13550-023-00955-w

**Published:** 2023-01-31

**Authors:** Fuzhen Zeng, Jingwan Fang, Amanjule Muhashi, Huafeng Liu

**Affiliations:** State Key Laboratory of Modern Optical Instrumentation, College of Optical Science and Engineering, Zhejiang University, Hangzhou, China

**Keywords:** Dual-tracer PET, Reconstruction, Signal separation, Multi-task learning

## Abstract

**Background:**

Simultaneous dual-tracer positron emission tomography (PET) imaging can observe two molecular targets in a single scan, which is conducive to disease diagnosis and tracking. Since the signals emitted by different tracers are the same, it is crucial to separate each single tracer from the mixed signals. The current study proposed a novel deep learning-based method to reconstruct single-tracer activity distributions from the dual-tracer sinogram.

**Methods:**

We proposed the Multi-task CNN, a three-dimensional convolutional neural network (CNN) based on a framework of multi-task learning. One common encoder extracted features from the dual-tracer dynamic sinogram, followed by two distinct and parallel decoders which reconstructed the single-tracer dynamic images of two tracers separately. The model was evaluated by mean squared error (MSE), multiscale structural similarity (MS-SSIM) index and peak signal-to-noise ratio (PSNR) on simulated data and real animal data, and compared to the filtered back-projection method based on deep learning (FBP-CNN).

**Results:**

In the simulation experiments, the Multi-task CNN reconstructed single-tracer images with lower MSE, higher MS-SSIM and PSNR than FBP-CNN, and was more robust to the changes in individual difference, tracer combination and scanning protocol. In the experiment of rats with an orthotopic xenograft glioma model, the Multi-task CNN reconstructions also showed higher qualities than FBP-CNN reconstructions.

**Conclusions:**

The proposed Multi-task CNN could effectively reconstruct the dynamic activity images of two single tracers from the dual-tracer dynamic sinogram, which was potential in the direct reconstruction for real simultaneous dual-tracer PET imaging data in future.

## Introduction

Positron emission tomography (PET) is a nuclear imaging technique that uses radiolabeled tracers to measure metabolic functions in vivo. It provides useful information and guidance for the detection, diagnosis, staging, treatment planning and monitoring of diseases [1, 2].

For some diseases, a PET scan with a single tracer is insufficient to reveal the complex pathological characteristics. Researchers tried out multi-tracer PET imaging for more complete information, which was usually achieved by multiple single-tracer scans. For example, Fu et al. [3] demonstrated that combining the imaging results of $^{18}$F-fluorodeoxyglucose ($^{18}$F-FDG) and $^{18}$F-fluorocholine ($^{18}$F-FCH) led to more accurate detection of low-grade glioma than using $^{18}$F-FDG or $^{18}$F-FCH only.

Instead of two separate single-tracer scans, a simultaneous dual-tracer PET scan can eliminate errors caused by image misalignment and physiological changes between scans, and also reduce the total scanning time. However, different tracers all generate the 511-keV photon pairs, which makes signal separation a difficulty in the reconstruction for simultaneous dual-tracer PET imaging. Efforts have been devoted to the development of various separation techniques, including traditional methods mainly based on estimation and more recent approaches based on deep learning or machine learning.

Traditional estimation approaches have been extensively studied [4]. The earliest simualtion study [5] and later animal study [6] achieved signal separation based on different half-lives of two tracers. Later on, the parallel compartment model [7, 8] was widely used in tracer separation, by which the kinetic parameters of two tracers were simultaneously estimated to recover the activity distributions of each tracer. Several studies have investigated the effects of tracer combination, injection interval, and scanning protocol on the results of compartment model-based signal separation [9–12]. The model-based separation method was simplified by introducing a reference region [13] or the technique of reduced parameter space [14–16]. A state-space representation based on the compartment model [17] was proposed for simultaneous signal separation and reconstruction. In addition, separation methods based on principal component analysis [18], generalized factor analysis [19], or basis function fitting [20] were also proposed. In particular, Andreyev and Celler [21] proposed a physical method to distinguish different tracer signals using a specially labeled tracer that can additionally emit a high-energy $\gamma$ photon. Fukuchi et al. used special detectors to receive prompt $\gamma$-rays, and proposed reconstruction methods based on data subtraction for dual-tracer PET imaging when one tracer was labeled by a pure positron emitter and the other by a positron-$\gamma$ emitter [22, 23].

Since these traditional methods explicitly use specific prior information, they also have limitations in applications. Methods based on the difference of half-lives are not applicable to tracers with same or close half-lives. Methods based on the parallel compartment model require staggered injection and arterial blood sampling. To use compartment model with reference tissue method, the firstly injected tracer must have a reference region. Besides, component analysis may have non-unique solutions, especially if the component factors of the tracers are similar.

Compared to traditional methods, deep learning or machine learning methods for dual-tracer separation or reconstruction are not constrained by prior information, thus can deal with simultaneously injected tracers or tracers with the same half-lives.

There are two kinds of deep learning approaches for dual-tracer reconstruction: the indirect ones and direct ones. Indirect methods firstly reconstruct the dual-tracer dynamic images by traditional reconstruction algorithms, then separate the dual-tracer images by deep neural networks to obtain single-tracer images [24–28]. These networks separate signals either from the voxel time-activity curves (TACs) [24–27] or from the entire dynamic image [28], and are easily influenced by the quality of reconstructed images. Direct methods reconstruct images of two tracers directly from the dual-tracer sinogram, which means the network learns to solve both the separation task and reconstruction task. As an example, Xu and Liu [29] imitated a filtered back-projection (FBP) procedure by network to reconstruct dual-tracer images and used a three-dimensional (3D) convolutional neural network (CNN) to provide an estimation of two tracers from the reconstructed images, referred to as FBP-CNN model. FBP-CNN behaved better than indirect reconstruction methods since it also learned spatial information from sinogram. However, the FBP part of the model included tremendous number of parameters, causing demands for both large amount of training data and memories. The development of a more trainable and memory-friendly network is necessary. Apart from these deep learning models, the recurrent extreme gradient boosting, a machine learning algorithm, was recently used to separate tissue TACs in the $^{18}$F-FDG/$^{68}$Ga-DOTATATE imaging of neuroendocrine tumors [30].

This study aims to directly reconstruct activity distributions of two tracers from the dual-tracer sinogram by a novel deep neural network named Multi-task CNN, which is characteristic of:

(a) A 3D encoder-decoder structure. The deep encoder-decoder structure is motivated by DeepPET [31] which was used for single-tracer, static PET image reconstruction. To cope with the dynamic data in dual-tracer PET imaging, we adopt 3D convolution layers to form the encoder and decoder, by which the temporal kinetic features and spatial structural features are learned simultaneously. The former plays an important role in the separation task, while the latter is useful for reconstruction.

(b) A multi-task learning framework. The multi-task learning [32] forces a model to learn multiple different but related tasks simultaneously. The learned information is shared between tasks to improve the performances of the model. It has been widely used in computer vision [33] and medical image analysis [34]. In dual-tracer PET imaging, the reconstruction of two single-tracer activity images can be regarded as two different but related tasks. In Multi-task CNN, we set one common encoder while extend the decoder part into two branches.

(c) The direct encoding of sinogram. Unlike FBP-CNN which reconstructed the dual-tracer activity images by network before encoding, the Multi-task CNN avoids the reconstruction of dual-tracer images but directly encodes the dual-tracer sinogram.

## Methods

### Models of dual-tracer PET imaging and reconstruction

Generally, the process of a single-tracer PET imaging is formulated as

1 $$\begin{aligned} y(t) = Gx(t)+n(t), \end{aligned}$$

where *y*(*t*) and *x*(*t*) are the sinogram and activity distribution at time *t*. The system matrix *G* describes the probability of each voxel in the activity distribution to be detected by each bin in the sinogram, which is related to the physical and geometrical structure of the PET scanner [35]. And *n*(*t*) is the noise mainly caused by random and scattered coincidences.

The dual-tracer PET imaging with simultaneous injection can be defined as

2 $$\begin{aligned} y^{I+II}(t) = G\left[ x^I(t)+x^{II}(t)\right] +n^I(t)+n^{II}(t), \end{aligned}$$

where $y^{I+II}(t)$ is the dual-tracer sinogram, $x^I(t)$ and $x^{II}(t)$ are the activity distributions of Tracer I and Tracer II. The different spatiotemporal distributions of the tracers lead to different noises $n^I(t)$ and $n^{II}(t)$.

For a certain scanning protocol, the dynamic dual-tracer sinogram is denoted as $Y^{I+II}=[y^{I+II}(t_1),\dots ,y^{I+II}(t_F)]$, where *F* is the number of frames, $t_f$ is the mid-frame time of the $f^{th}$ frame. The dynamic single-tracer activity images can be denoted in the same way as $X^I$ and $X^{II}$. The direct reconstruction for dual-tracer PET imaging can be described as

3 $$\begin{aligned}{}[{\hat{X}}^I,{\hat{X}}^{II}]=h_\theta (Y^{I+II}), \end{aligned}$$

where ${\hat{X}}^I$ and ${\hat{X}}^{II}$ are the estimatation of $X^I$ and $X^{II}$. In this study, $\theta$ are the weights of the proposed Multi-task CNN.

### Multi-task CNN

#### Network architecture

**Fig. 1:**
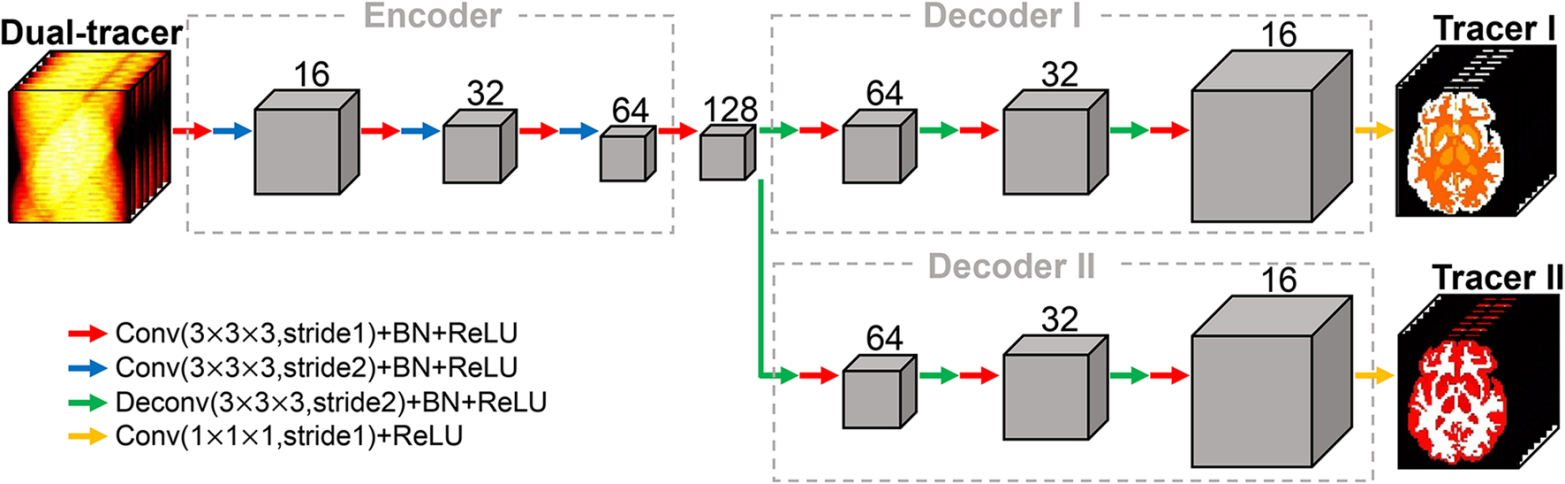
The architecture of Multi-task CNN. The dual-tracer dynamic sinogram is inputted to the encoder, and two decoders output the dynamic activity images of two tracers. Conv = convolution layer, BN = batch normalization layer, ReLU = rectified linear unit layer, Deconv=deconvolution layer. The kernel size and number of channels are noted in the figure

The architecture of Multi-task CNN is presented in Fig. 1. The dual-tracer dynamic sinogram of a single slice, in the shape of frames $\times$ bins $\times$ angles, is taken in as the network input. The spatiotemporal features are extracted from the dynamic sinogram by the encoder, which includes three downsampling blocks. In each block, a 3D convolution layer with a stride of 1 is firstly applied, with the size of each feature map maintained. Then, the second 3D convolution layer downsamples the features by a stride of 2. The number of channels in three blocks are 16, 32 and 64. At the end of the encoder, a 3D convolution layer is used to extend the features to 128 channels.

The multi-task learning scheme is reflected in using two decoders to estimate the activity images of two tracers separately. The two decoders have the same structure, both including three upsampling blocks. In each block, a 3D deconvolution layer is firstly used to upsample the features by a stride of 2. Then a 3D convolution layer with a stride of 1 is applied, with the size of features kept unchanged. Symmetry to the encoder, the number of channels are 64, 32, 16 in these blocks. Finally, a 3D convolution layer with a stride of 1 is used to reduce the number of channels to 1. All the convolution and deconvolution layers are connected by a batch normalization layer and a rectified linear unit (ReLU) layer. The size of the kernels are $3 \times 3 \times 3$ in all convolution and deconvolution layers, except the last layer which uses 1 $\times$ 1 $\times$ 1 kernels.

#### Loss function

The loss function of a single-frame image of one tracer is a combination of the mean squared error (MSE) and the structural similarity (SSIM) [36]:

4 $$\begin{aligned} L({\hat{x}},x)= \alpha {\text{MSE}}({\hat{x}},x)-\beta \ln {\frac{1+{\text{SSIM}}({\hat{x}},x)}{2}}, \end{aligned}$$

where ${\hat{x}}$ is an estimated single-frame activity image, and *x* is the corresponding label. $\alpha$ and $\beta$ are the coefficients that balance MSE and SSIM.

The total loss $L_{\text{total}}$ was summed by the loss of two tracers and averaged by frames:

5 $$\begin{aligned} L_{\text{total}}=\frac{1}{F}\sum _{f=1}^F L\left[ {\hat{x}}^I(t_f),x^I(t_f)\right] +L\left[ {\hat{x}}^{II}(t_f),x^{II}(t_f)\right] . \end{aligned}$$

### Comparative method and evaluation metrics

The proposed Multi-task CNN was compared to FBP-CNN [29] by MSE, multiscale structural similarity (MS-SSIM) index [37] and peak signal-to-noise ratio (PSNR). Frame-wise MS-SSIM is formulated as

6 $$\begin{aligned} {\text{MS}-{\text{SSIM}}({\hat{x}},x)}=[l_M({\hat{x}},x)]^{\gamma _{{1},M}}\cdot \prod _{m=0}^M[c_m ({\hat{x}},x)]^{\gamma _{{2},m}}[s_m({\hat{x}},x)]^{\gamma _{{3},m}}, \end{aligned}$$

where *M* represents the number of dowmsampling operations by a factor of 2 performed to the estimated image ${\hat{x}}$ and label image *x*. The subscript *m* is related to the $m^{th}$ downsampling operation. For instance, $c_m({\hat{x}},x)$ calculates $c(\cdot ,\cdot )$ of the *m*-times downsampled images, and its corresponding exponential parameter is $\gamma _{2,m}$. $l({\hat{x}},x)$, $c({\hat{x}},x)$ and $s({\hat{x}},x)$ measure the differences of luminent, contrast and structure similarity between two images. The detailed calculation of $l(\cdot ,\cdot )$, $c(\cdot ,\cdot )$ and $s(\cdot ,\cdot )$, as well as the values of *M* and exponential parameters can refer to the previous publication [37]. And the frame-wise PSNR is formulated as:

7 $$\begin{aligned} {\text{PSNR}}({\hat{x}},x)=10\cdot \log _{10}\left[ \frac{x_{\max }^2}{\text{MSE}({\hat{x}},x)}\right] , \end{aligned}$$

where $x_{\max }$ is the maximum value of *x*.

## Experiments

In this study, we trained and tested the Multi-task CNN and FBP-CNN on four simulated datasets and one animal dataset from real PET experiments.

### Simulation study

#### Experimental settings

**Table 1:** Settings of the simulation experiments

EXP	Tracers	Scanning protocol	Kinetic variation (%)	Input variation (%)	Parameter sets
1-1	$^{11}$C-FMZ/$^{11}$C-acetate	30s$\times$4+110s$\times$12+180s$\times$2	10	10	30
1-2	$^{11}$C-FMZ/$^{11}$C-acetate	30s$\times$4+110s$\times$12+180s$\times$2	20	10	30
2	$^{11}$C-FMZ/$^{11}$C-acetate	30s$\times$4+110s$\times$12+180s$\times$2	10	10	15
	$^{18}$F-FDG/$^{11}$C-FMZ	60s$\times$2+180s$\times$6+240s$\times$10	10	10	15
3	$^{18}$F-FDG/$^{11}$C-FMZ	60s$\times$2+90s$\times$2+150s$\times$14	10	10	10
	$^{18}$F-FDG/$^{11}$C-FMZ	60s$\times$3+140s$\times$7+230s$\times$8	10	10	10
	$^{18}$F-FDG/$^{11}$C-FMZ	60s$\times$2+180s$\times$6+240s$\times$10	10	10	10

The simulation study contained four experiments: two individual difference experiments (EXP 1-1, EXP 1-2), one tracer combination experiment (EXP 2) and one scanning protocol experiment (EXP 3). The detailed settings are shown in Table 1. To simulate the physiological characteristics of different people, we applied Gaussian randomization to the tracer kinetic parameters and parameters of plasma input function in all experiments. The numbers of parameter sets (including both kinetic parameters and input function parameters) and the levels of physiological variation are listed in Table 1. In all experiments, the number of frames of the dynamic data was set to 18.

Exp 1-1 and EXP 1-2 simulated data of 30-min $^{11}$C-Flumazenil ($^{11}$C-FMZ)/$^{11}$C-acetate imaging. The two experiments designed the same variation range (10%) of input functions but different variation ranges (10%, 20%) of kinetic parameters. In EXP 2, we set two combinations of tracers, where $^{11}$C-FMZ/$^{11}$C-acetate had the same half-life, while $^{18}$F-FDG/$^{11}$C-FMZ had different half-lives. The scanning protocols were different accordingly. The levels of both input variation and kinetic variation of two combinations were set to 10%. In EXP 3, we designed three scanning protocols with the durations of 40 min, 50 min and 60 min for $^{18}$F-FDG/$^{11}$C-FMZ imaging. All the variation ranges were set to 10%.

#### Simulation process

**Fig. 2:**
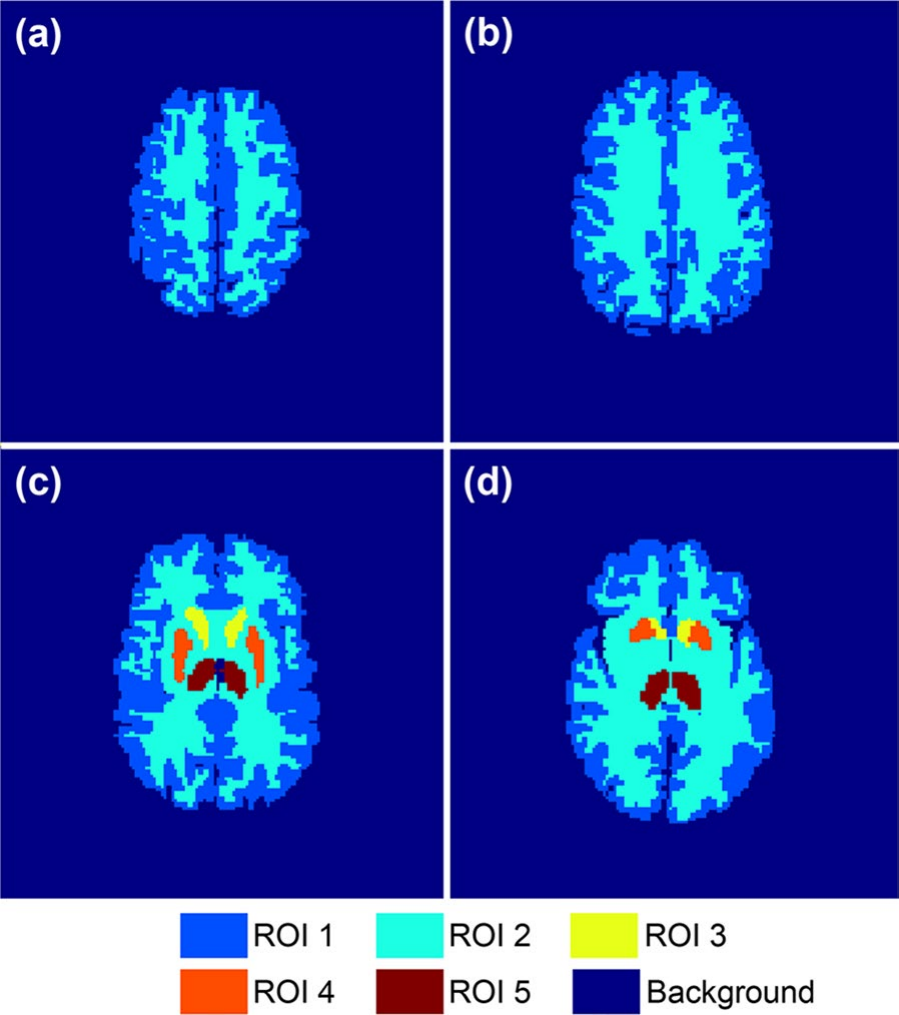
The Zubal phantoms used in simulation experiments. **a** **b**, **c**, and **d** are representative phantoms corresponding to differenct slices of the original 3D phantom

The phantoms used for simulation were originated from the 3D Zubal brain phantom [38]. By region of interest (ROI) partition, slice selection and dowmsampling, we obtained 40 two-dimensional phantoms sized 128 $\times$ 128. The voxel size was $2.2 \times 2.2 \times 1.4$ mm$^3$. The number of ROIs in each phantom varied from 2 to 5. Representative phantoms are shown in Fig. 2.

The single-tracer dynamic activity images were generated based on the two-tissue compartment model [39] which described the kinetic characteristics of the tracer:

8 $$\begin{aligned} \begin{aligned} \dfrac{\text{d}C_1(t)}{\text{d}t}&=K_1 C_P(t)-(k_2+k_3)C_1(t)+k_4 C_2(t) \\ \dfrac{\text{d}C_2(t)}{\text{d}t}&=k_3 C_1(t)-k_4 C_2(t) \\ C_T(t)&=C_1(t)+C_2(t) \end{aligned} \end{aligned}$$

$C_1(t)$ and $C_2(t)$ are the tracer concentrations in two tissue compartments. The two compartments represent different metabolic states of the tracer. $C_P(t)$ is the tracer concentration in plasma, i.e., the plasma input function. $K_1$, $k_2$, $k_3$ and $k_4$ are the kinetic parameters reflecting the speed of directional tracer exchanges between different tissue compartments or between plasma and tissue, which are different among ROIs, individuals and tracers. The input functions of $^{18}$F-FDG [40], $^{11}$C-FMZ [41] and $^{11}$C-acetate [18] were generated by different models. By applying Gaussian randomization to the kinetic parameters and parameters of input function, we simulated individual differences in physiological states. The mean values of these parameters were set as previously reported [7, 15, 18, 40–42], and the standard deviations were set as a portion of mean values, which are shown in Table 1. After setting the aforementioned phantoms, parameters and the scanning protocols, the tissue TACs $C_T(t)$ were obtained by numerically solving Eq. (8) by the COMKAT toolbox [43]. All voxel TACs formed the noise-free dynamic activity images. The images generated were sized 128 $\times$ 128 $\times$ 18 frames.

The single-tracer dynamic sinograms were generated by frame-wise projection of single-tracer activity images using Michigan Image Reconstruction Toolbox [44]. We designed the system matrix by simple strip integrals according to the geometry of the Siemens Inveon PET/CT scanner [45], without the consideration of attenuation, scatter and variations in detector efficiencies. We added 20% random noise and Poisson noise to the sinograms. The size of the dynamic sinogram was 128 bins $\times$ 160 angles $\times$ 18 frames.

The dual-tracer dynamic sinogram was obtained by simply adding up two single-tracer sinograms. This guaranteed the alignment of images, the match of doses and the consistency of physiological states between the dual-tracer scan and two single-tracer scans. The dual-tracer dynamic sinogram was used as the network input.

Additionally, the single-tracer dynamic activity images with noise were reconstructed frame-wisely by the ordered subsets expectation maximization (OSEM) algorithm [46] with 6 iterations and 5 subsets. Corrections and normalization were not performed. In consistence with the phantom and simulated noise-free activity images, the voxel size of the reconstructed images was $2.2 \times 2.2 \times 1.4$ mm$^3$. The reconstructed images of two tracers were used as labels.

#### Datasets and network training

Datasets used in EXP 1-1, EXP 1-2, EXP 2 and EXP 3 all included 30 parameter sets. In particular, 15 sets of parameters were simulated for each one of the two tracer combinations in EXP 2, and 10 sets of parameters were simulated for each one of the three scanning protocols in EXP 3. Therefore, the four datasets used for network training all contained 1200 groups (40 phantoms $\times$ 30 parameter sets) of simulated PET data. Each dataset was randomly divided into training data, validation data and test data by 8:1:1. The inputs were normalized by the mean and standard deviation of the training set, and the labels were normalized by dividing 255.

We used the Adam optimizer in the training of Multi-task CNN and FBP-CNN. For Multi-task CNN, the hyperparameters were set as: learning rate = 0.0005, epochs = 100, batch size = 4, $\alpha$ = 100 and $\beta$ = 1. For FBP-CNN, the hyperparameters were set as: learning rate = 0.0001, epochs = 100 and batch size = 4.

### Animal study

#### Animals and tracers

The animal experiments were approved by the Experimental Animal Ethics Committee of Southern Medical University, and were conducted in compliance with the ARRIVE guidelines, the guidelines of the US National Institutes of Health and local legal requirements. 13 SD rats (average age: 9 weeks; average weight: 300 g) with a C6 cell intracranial orthotopic xenograft glioma model were used in this experiment.

Previous PET study showed that $^{18}$F-FDG had a higher sensitivity but a lower specificity than *O*-(2-[$^{18}$F]fluoroethyl)-L-tyrosine ($^{18}$F-FET) in the detection of head and neck squamous cell carcinoma [47]. A $^{18}$F-FDG/$^{18}$F-FET dual-tracer PET scan might be benificial for better detection and location of tumors. We selected $^{18}$F-FDG and $^{18}$F-FET as tracers to evaluate the feasibility of our model on real PET data.

#### PET imaging

Two single-tracer PET scans were performed on each rat using a Siemens Inveon PET/CT scanner [45]. The rats underwent an 8-hour fasting before PET scans. During the scans, they were kept under anesthesia by 1% isoflurane and 1 L/min of oxygen, and also fixed by medical tapes. At the beginning of each scan, $^{18}$F-FDG or $^{18}$F-FET was injected at a dose around 37 MBq into the tail vein of the rat. The $^{18}$F-FDG scan and $^{18}$F-FET scan followed the same scanning protocol (60 s $\times$ 10, 300 s $\times$ 3420 s $\times$ 5), and were carried out on two different days in random order.

#### Data preparation

The 3D dynamic sinogram of each single-tracer scan was obtained. Using the built-in software, the dynamic activity images were reconstructed frame-wisely by 3D-OSEM algorithm with normalization and random correction. Attenuation correction was also performed using individual computed tomography (CT) images. The reconstructed single-tracer activity images were sized 128 $\times$ 128 $\times$ 18 frames with a voxel size of $0.78 \times 0.78 \times 0.80$ mm$^3$, and were used as labels.

Since the network input should be a time sequence of a 2D sinogram, the original 3D sinogram was random corrected and re-organized by single slice rebinning to form 2D sinogram. The obtained dynamic sinograms were sized 128 bins $\times$ 160 angles $\times$ 18 frames. As done in previous dual-tracer PET experiments [14, 48], we added sinograms of two tracers to get the dual-tracer sinogram, which was used as the network input.

#### Datasets and network training

Totally, 351 brain slices from 13 rats were selected, among which 297 groups of data from 11 rats were used for training and validation, the remaining 54 groups of data from the other 2 rats were used for testing. The inputs were normalized by the mean and standard deviation of the training set, and the labels were normalized by dividing $10^6$, which converted the unit from Bq/mL to MBq/mL.

Same as the simulation study, Adam optimizer was used in the training of Multi-task CNN and FBP-CNN. For Multi-task CNN, the hyperparameters were set as: learning rate = 0.0001, epochs = 100, batch size = 4, $\alpha$ = 1 and $\beta$ = 0.01. Moreover, a weight decay with a coefficient of 0.01 was added to the loss function. For FBP-CNN, the hyperparameters were set as: learning rate = 0.00002, epochs = 250, batch size = 4.

## Results

### Simulation study

**Table 2:** Quantitative results of the simulation experiments

EXP	Dataset	Method	Tracer I			Tracer II		
			MSE	MS-SSIM	PSNR	MSE	MS-SSIM	PSNR
1-1	Individual difference I	FBP-CNN	2.3561	0.8783	23.92	6.1724	0.9248	30.32
		Proposed	**1.2661**	**0.9534**	**27.61**	**1.8536**	**0.9927**	**35.85**
1-2	Individual difference II	FBP-CNN	4.1080	0.8586	22.21	11.4220	0.9139	26.67
		Proposed	**1.4566**	**0.9592**	**27.47**	**4.5473**	**0.9889**	**33.57**
2	Tracer combination I	FBP-CNN	1.5350	0.8222	23.86	7.5759	0.9231	30.00
		Proposed	**0.3599**	**0.9705**	**30.02**	**2.7090**	**0.9897**	**34.41**
	Tracer combination II	FBP-CNN	4.8387	0.8956	24.32	2.9304	0.4762	17.13
		Proposed	**0.6687**	**0.9872**	**32.77**	**0.2906**	**0.9561**	**28.52**
3	Scanning protocol I	FBP-CNN	2.9040	0.8555	24.63	1.2512	0.8592	23.81
		Proposed	**0.4325**	**0.9787**	**31.94**	**0.2508**	**0.9789**	**30.51**
	Scanning protocol II	FBP-CNN	2.8501	0.8560	24.75	1.1370	0.8691	24.40
		Proposed	**0.4504**	**0.9778**	**31.78**	**0.2753**	**0.9759**	**29.75**
	Scanning protocol III	FBP-CNN	3.1287	0.8539	24.71	1.2879	0.8116	21.16
		Proposed	**0.4722**	**0.9799**	**32.28**	**0.2476**	**0.9676**	**28.58**

The lower MSE, higher MS-SSIM and higher PSNR are noted in bold font in the comparison of the proposed method and FBP-CNN

Table 2 lists the mean values of MSE, MS-SSIM and PSNR of each test datasets. In all simulation experiments and datasets, Multi-task CNN showed lower MSE, higher MS-SSIM and PSNR than FBP-CNN. The results of each simulation experiment are as follows.

#### Effect of individual difference

**Fig. 3:**
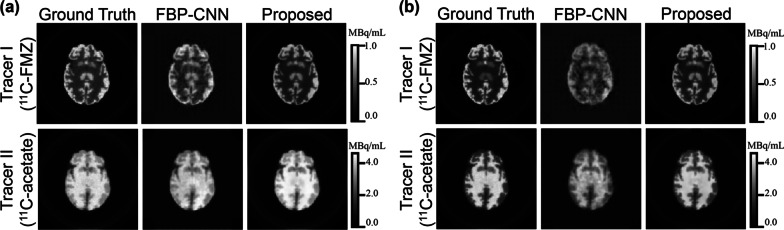
The representative reconstructed images from the individual difference experiment. **a** 10% kinetic variation, **b** 20% kinetic variation. These images show the 10th frame of the dynamic images

As shown in Fig. 3, single-tracer activity images reconstructed by Multi-task CNN contained less noise than those reconstructed by FBP-CNN, and were even smoother than the ground truth in some regions. When the kinetic variation increased from 10% (Fig. 3a) to 20% (Fig. 3b), the reconstruction results of FBP-CNN became worse, where the image details were not well reconstructed.

As displayed in Table 2, for both tracers under two levels of individual difference, the Multi-task CNN reconstructions had significantly lower MSE, higher MS-SSIM and PSNR than FBP-CNN reconstructions. When the level of individual difference increased, the MSE of both methods increased, while Multi-task CNN increased by a smaller increment. The MS-SSIM and PSNR of FBP-CNN reconstructions of two tracers all decreased. Although the MS-SSIM and PSNR of Multi-task CNN reconstructions of Tracer II ($^{11}$C-acetate) were also slightly reduced, the two-tailed t tests showed that the MS-SSIM (${T}=-2.57, {P}=0.011$) and PSNR (${T}=0.42, {P}=0.674$) of reconstructed images of Tracer I ($^{11}$C-FMZ) were not influenced.

#### Effect of tracer combination

**Fig. 4:**
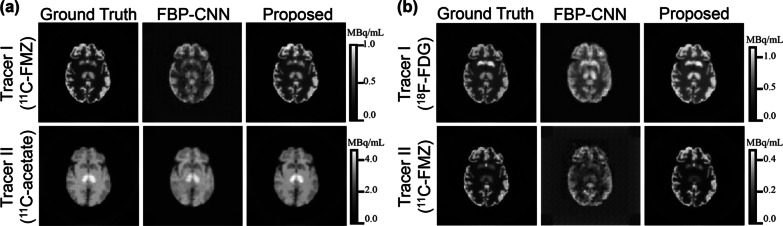
The representative reconstructed images from the tracer combination experiment. **a** Tracer combination I ($^{11}$C-FMZ/$^{11}$C-acetate), **b** Tracer combination II ($^{18}$F-FDG/$^{11}$C-FMZ). These images show the 10th frame of the dynamic images

Visually in Fig. 4, Multi-task CNN reconstructed images with better details and less noise than FBP-CNN. Table 2 quantitatively shows that the MS-SSIM and PSNR of Multi-task CNN reconstructions were higher, and the MSE was lower.

It is worth noting that $^{11}$C-FMZ was set as Tracer I in the first tracer combination (Fig. 4a), but as Tracer II in the second combination (Fig. 4b). In both cases, $^{11}$C-FMZ images reconstructed by FBP-CNN showed an “checkerboard effect”, which was more obvious in the second combination, resulting in extremely low MS-SSIM (0.4762 ± 0.0475) and PSNR (17.13 ± 0.33). Compared to FBP-CNN, metrics of Multi-task CNN reconstructions were more stable between different tracer combinations.

#### Effect of scanning protocol

**Fig. 5:**
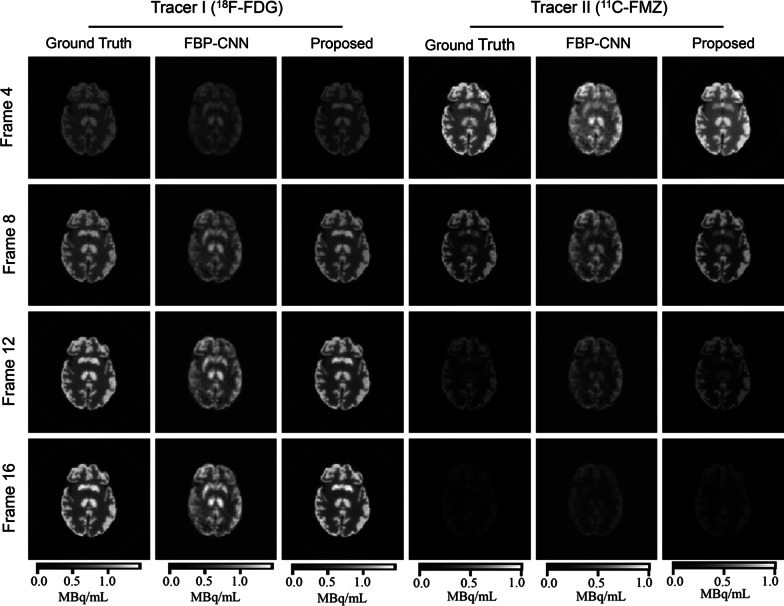
The representative reconstructed images from Protocol III (60-min scan) of the scanning protocol experiment

Figure 5 shows the reconstructed images of $^{18}$F-FDG/$^{11}$C-FMZ under one of the scanning protocols. The 60-min scan was too long for $^{11}$C-FMZ imaging, causing quite different concentrations of two tracers in later frames. Even in such case, both Multi-task CNN and FBP-CNN can well reconstructed the temporal changes of tracer activities. However, the structures of $^{18}$F-FDG images reconstructed by FBP-CNN were inconsistent with the ground truth. Furthermore, in the 16th frame of $^{11}$C-FMZ, both methods overestimated the tracer activities, while the estimation of Multi-task CNN was closer to the label.

**Fig. 6:**
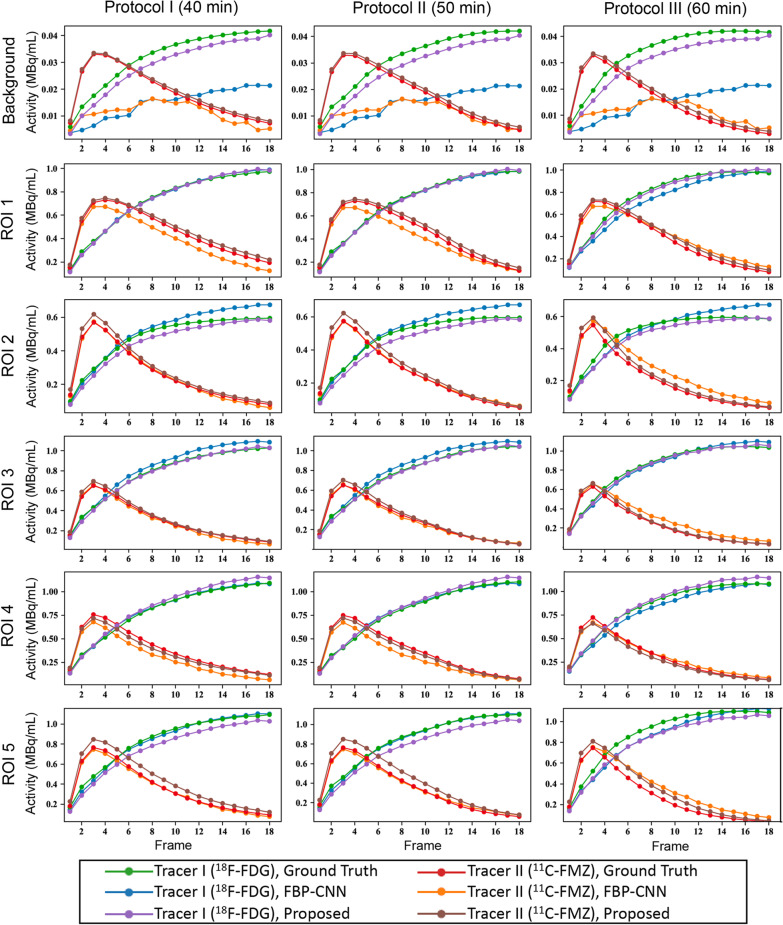
The ROI-TACs from the scanning protocol experiment

Overall, MS-SSIM and PSNR of Multi-task CNN reconstructions were higher than FBP-CNN, and the MSE was lower (Table 2). Figure 6 shows the ROI-TACs extracted from the reconstructed images. Each subplot contains estimated and label TACs of two tracers. The rows and columns of the figure represent different ROIs and scanning protocols. Obviously shown in the first row, TACs of FBP-CNN reconstructions severely deviated from the labels, which was resulted from negative values in the background. The TACs from Multi-task CNN reconstructions were closer to the label TACs in the background and ROI 1-4, while TACs from FBP-CNN reconstructions were more accurate in ROI 5. Besides, changes in scanning protocols had little influence on the performance of both methods.

### Animal study

**Fig. 7:**
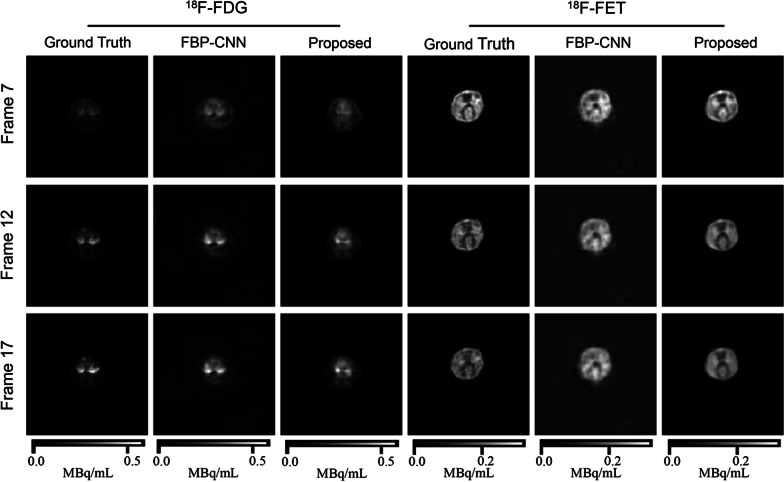
The representative reconstructed images from the animal experiment. These images show the same slice of one rat

**Table 3:** Quantitative results of the animal experiment

Metric	Tracer	Method	Frame 4	Frame 8	Frame 12	Frame 16
MSE	$^{18}$F-FDG	FBP-CNN	0.0004	0.0003	0.0004	**0.0004**
		Proposed	** 0.0003**	** 0.0003**	** 0.0003**	0.0004
	$^{18}$F-FET	FBP-CNN	0.0014	0.0013	0.0011	0.0010
		Proposed	** 0.0008**	** 0.0007**	** 0.0006**	** 0.0006**
MS-SSIM	$^{18}$F-FDG	FBP-CNN	0.8193	0.8822	0.9080	**0.9115**
		Proposed	**0.8719**	**0.8993**	**0.9113**	0.9055
	$^{18}$F-FET	FBP-CNN	0.7549	0.7532	0.7755	0.7863
		Proposed	**0.8884**	**0.8787**	**0.8765**	**0.8727**
PSNR	$^{18}$F-FDG	FBP-CNN	22.84	25.67	27.76	28.56
		Proposed	**24.66**	**26.53**	**28.17**	**28.59**
	$^{18}$F-FET	FBP-CNN	24.44	23.68	23.91	23.67
		Proposed	**27.49**	**26.90**	**26.51**	**26.00**

The lower MSE, higher MS-SSIM and higher PSNR are noted in bold font in the comparison of the proposed method and FBP-CNN. When calculating MSE, activity unit was converted to MBq/mL

Figure 7 shows the representative images of the same brain slice of one rat. The $^{18}$F-FDG images reconstructed by both methods were less-accurate. As for $^{18}$F-FET images, the results of Multi-task CNN showed better details and contrast, and the tumor near the top could also be distinguished. Table 3 also shows that images reconstructed by Multi-task CNN had lower MSE, higher MS-SSIM and PSNR than FBP-CNN. Compared with the simulation experiments, the qualities of reconstructed images in animal experiment were significantly worse, which was mainly caused by misalignment of images from two single-tracer scans.

## Discussion

Most traditional reconstruction methods for dual-tracer PET imaging could only separate two tracers when they were administrated with an interval, and were sensitive to tracer combinations and scanning protocols. These methods usually required invasive measurements of plasma input function by arterial blood sampling. Compared to the traditional methods, the proposed Multi-task CNN as well as other deep learning-based models could effectively separate signals from simultaneoulsly administrated tracers, and avoid the arterial blood sampling.

The deep learning-based indirect reconstruction models separate the dual-tracer images after reconstruction. The majority of these methods separate signals voxel-wisely, that is, by separating the TACs extracted from the reconstructed images [24–27]. Only temporal features were learned from the TACs and used for separation. There also existed a method separating from the image domain, using both spatial and temporal features [28]. However, these two types of methods were both influenced by the quality of reconstructed images. As a direct reconstruction method, the Multi-task CNN extracted and fully used the spatiotemporal information from the dual-tracer sinogram, which was not influenced by the traditional reconstruction algorithms.

In the current study, we quantitatively compared our Multi-task CNN to FBP-CNN [29], which is also a deep neural network for direct reconstruction for dual-tracer PET imaging. The robustness of Multi-task CNN and FBP-CNN to changes in tracer combiantion and scanning protocol was evaluated by simulation data. To make the simulated data more realistic, we used phantoms corresponding to different brain slices, and generated data with varied tracer kinetic parameters and input functions to imitate individual difference. Whether the models were sensitive to different levels of individual difference was also studied.

According to the results of simulation experiments, Multi-task CNN reconstructed single-tracer activity images with higher quality than FBP-CNN, and was more robust to individual difference and tracer combination than FBP-CNN. In the animal experiment, Multi-task CNN also showed its superiority over FBP-CNN.

The advantages of Multi-task CNN over FBP-CNN might be mainly due to different scales of two models. FBP-CNN explicitly consisted of two parts. Firstly, the reconstruction part unrolled the FBP algorithm by a convolution layer and a fully-connected layer to reconstruct the dual-tracer activity image. Then, the separation part estimated single-tracer images by 3D encoders and decoders. The number of parameters in the reconstruction part, especially the fully-connected layer, growed with the size of sinogram and activity image, which resulted in low generalization ability of the model. Unlike FBP-CNN, the encoder was directly operated on the sinogram in Multi-task CNN. The number of parameters in Multi-task CNN was fixed and independent of the size of sinogram or image. Nevertheless, the encoder part of the Multi-task CNN was less interpretable than the reconstruction part of the FBP-CNN.

The effectiveness of multi-task learning could be explained in two perspectives [32]. From the perspective of overfitting, it was commonly agreed that learning more than one task could prevent the model from overfitting, since it was more challenging than learning a single task. From the perspective of auxiliary learning, learning related tasks was beneficial for the model to extract appropriate and important features. In the Multi-task CNN, the reconstruction of two single-tracer activity images were regarded as two tasks. The two tasks had the same scanning protocol and system probability matrix since they were scanned simultaneously. However, the two tracers had different kinetic characteristics, input functions, as well as different levels of noise due to different activity distributions. Using Multi-task CNN could learn more substantial and important features by the encoder, and maintain the differences between two tracers by using two decoders.

However, the current study had several limitations. As shown in Fig. 6, in some ROIs, the activities reconstructed by the proposed method deviated from the ground truth. The TACs of $^{11}$C-FMZ tended to be inaccurate in early frames, while TACs of $^{18}$F-FDG were inaccurate in later frames. The introducing of attention mechanism to the model might be helpful to better extract different features from different frames. In addition, the images reconstructed by Multi-task CNN were over smoothed, which might be due to including SSIM in the loss function. A more appropriate coefficient of SSIM part in the loss function, and more suitable constraints should be further studied. Moreover, the dual-tracer sinograms in animal experiments were not obtained from dual-tracer scans, but from single-tracer scans for simplication, where the images were not aligned.

To use real experimental or clinical data to train deep learning models in future studies, standards of data acquicision and preprocessing should be set up. The prototols of simultaneous dual-tracer PET scans, and the issues concerning image alignment and dose matching need special focuses. For example, the injected doses of tracers in both single-tracer scans and dual-tracer scan are needed for scaling and dose matching. Images intra- and inter- scans should be well-aligned. The decay correction is not necessary since the concentrations of tracers in tissues are unknown. And the scanning protocol, corrections and reconstruction should be kept consistent between scans.

## Conclusions

In this study, we proposed Multi-task CNN, a 3D encoder-decoder network based on multi-task learning, to directly reconstruct two single-tracer dynamic images from the dual-tracer dynamic sinogram. The proposed method was superior to the existing FBP-CNN, exhibiting its robustness to individual difference, tracer combination and scanning protocol in the simulation study. In the animal study, the feasibility of applying this method to real PET data was preliminarily verified. The proposed Multi-task CNN was thus considered a potential deep learning-based method for the direct reconstruction of simultaneous dual-tracer PET imaging.

## Data Availability

The datasets used or analysed during the current study are available from the corresponding author on reasonable request.

## Abbreviations

PET: Positron emission tomography
TAC: Time-activity curve
FBP: Filtered back-projection
3D: Three-dimentional
CNN: Convolutional neural network
ReLU: Rectified linear unit
MSE: Mean squared error
SSIM: Structural similarity
MS-SSIM: Multiscale structural similarity
PSNR: Peak signal-to-noise ratio
ROI: Region of interest
CT: Computed tomography
